# THE ASSOCIATION BETWEEN MULTIPLE CHRONIC CONDITIONS AND DEPRESSIVE SYMPTOMS: DISTINCTIONS BY RACE, GENDER, AND AGE

**DOI:** 10.1093/geroni/igz038.1148

**Published:** 2019-11-08

**Authors:** Christy L Erving, Cleothia Frazier

**Affiliations:** Vanderbilt University, Nashville, Tennessee, United States

## Abstract

Multiple chronic conditions (MCC)—the co-occurrence of two or more chronic diseases—is a serious concern due to its high prevalence among middle-age and older-adults, and its association with increased disability, mortality risk, and healthcare costs. A growing body of work has shown that chronic physical conditions are associated with depressive symptoms. While MCC and depression affect a substantial proportion of older adults in general, there are important status variations in disease burden along the dimensions of race, gender, and age. This study employs an intersectional and multi-hierarchical approach to assess how these status characteristics (race, gender, and age) may condition the MCC-depression association. We use data from the 1994-2014 waves of the Health and Retirement Study (HRS), a nationally representative data source providing a longitudinal survey of U.S. adults over the age of 50 biennially. Results revealed that MCC was positively associated with depression in general. However, Black Americans, women, and younger adults were more likely to experience depression relative to their White, male, and older counterparts, respectively. The findings suggest that the lived experience of MCC differs by social status, and is perhaps due (in part) to status differences in access to social resources to counteract the potentially deleterious psychological effects of MCC. This research has also has practical implications: given the strong MCC-depression association, older adults with MCC should be offered psychological services to decrease the likelihood of developing mental health problems due to the stress associated with having multiple chronic conditions.

